# Towards an enhanced interpretation of δ^18^O changes during the past 300 kyr over Asia

**DOI:** 10.1093/nsr/nwac219

**Published:** 2022-10-21

**Authors:** Gilles Ramstein

**Affiliations:** Laboratoire des Sciences du Climat et de l’Environnement, LSCE/IPSL, CEA-CNRS-UVSQ, Université Paris-Saclay, France

For many decades, the impact of orbital forcing on monsoon systems has been intensively studied. Rapidly, attention has been drawn to the Mid-Holocene due to the ‘Green Sahara’, which was depicted, using many different proxies, as much more humid than the present-day desert. It also corresponds to enhanced Asian monsoons [1]. Climate models have been used to simulate this period. At first, only atmospheric models [2] and EMICs (Earth models of intermediate complexity) [3] were used. Great efforts were made for the model/data comparison. Then, associated with the development of AOGCMs (atmosphere ocean general circulation models) and more reliable data synthesis, large intercomparison projects took place. A very important step was the development of PMIP (Paleoclimate Model Intercomparison Project) [4,5]. The Mid-Holocene period was always considered as a reference, due to its strong response to seasonal orbital forcing. These intercomparisons were extremely efficient and enabled analyses of the robust characteristics using models, but they also favored the development of multi-proxy databases and strengthened model/data comparisons.

The development of O^18^ water isotope enabled models [6,7] was another important step. However, the interpretation of the isotopic signals proved to be a difficult task [8].

The paper by Liu *et al*. [9] revisits this issue, not only for snapshot periods, but it enlightens the topic considerably by producing a transient simulation of 300 kyr, including several glacial/interglacial cycles. Moreover, this paper focuses on the Asian monsoons with a seasonal interpretation of precipitation δ^18^O (δ^18^O_p_) signals in three different regions, central arid Asia (CA), southern Asia (SA) and eastern Asia (EA). The most interesting part of this detailed analysis is that it demonstrates which processes are hidden by the annual δ^18^O_p_ variation. In particular, the analysis of these transient simulations shows that the forcing mechanisms are different over the three regions. While the common feature is the dominant role played by the precession cycle, the responses are driven by distinct processes over the three regions.

In CA, temperature and water transport are the main causes of the evolution of δ^18^O_p_, whereas in SA, with a rainy season extending from June to September, the intensification of monsoons and the upstream depletion effect explain most of the signal. For EA, the southwesterly water vapor transported during the late monsoon season, together with the water vapor from the western Pacific, due to the retreat of the Northern Hemisphere ice sheet, results in a mixture of frequencies (23 and 100 kyr).

This study is interesting because there are not many long simulations involving water isotope processes, because the required computational resource is huge, so the authors are obliged to overcome this issue by using acceleration techniques [10]. Moreover, even though the transient simulation length is only 300 kyr compared to the eccentricity cycle of 100 kyr, it allows an investigation into the influences of the glacial/interglacial cycles, especially on EA, where the loess records have depicted its impact. In this study, a low-resolution climate system model (the NCAR CSM version 3) [11] and the corresponding atmospheric model were used, with water isotope enabled and computed sea surface temperatures (SSTs) derived from the AOGCM simulation, to analyze the transient behavior of the δ^18^O_p_, and more importantly, enable attribution of the processes responsible for δ^18^O_p_ evolution.

As the first step, the authors clearly pinpoint the presence of the precession cycles with different responses for CA, SA and EA. To go further, they take advantage of the simulation of their transient ^18^O water isotope to disentangle the different processes responsible for this distinct behavior.

For the CA region, the modulation of troposphere temperature as well as the ^18^O during the rainy season explain the variation of δ^18^O_P_ at the orbital scale.

Regarding the behavior of δ^18^O_p_ in the SA region, the anticorrelation with precipitation points to the amount effect. The authors demonstrate that the amount effect as well as the upstream depletion effect are the main processes explaining the δ^18^O_p_ variation during the rainy season. Moreover, they show that the Indian monsoon has a very stable origin in the south tropical Indian Ocean through interglacial/glacial cycles.

The conclusions are quite different for the EA region. First, the annual signal in δ^18^O_p_ is not driven by the rainy season temperature or precipitation. The large dynamics of the transportation of water vapor are essential for understanding the δ^18^O_p_ evolution for different seasons. The authors demonstrate that it is mainly controlled by upstream convective activity or rainout processes during the late season (August–September). Another important point concerning EA is that the retreat of the ice sheet essentially implies a strengthening of water vapor transport from the western Pacific towards East Asia. Moreover, the authors depict different phase lags with the precession cycle for the three different regions.

On the one hand, the progress made here in terms of seasonal analysis makes it difficult to validate the findings depicted in this paper. On the other hand, it is in fact possible to enhance the details of the analysis for each grid point, disentangling all the different physical processes involved in the variation of δ^18^O_p_ and identifying the major contributors. This last point is crucial for a correct interpretation of δ^18^O_p_ recorded in speleothems, ice cores, or ostracods in lake deposits.
